# Application of high throughput in vitro metabolomics for hepatotoxicity mode of action characterization and mechanistic-anchored point of departure derivation: a case study with nitrofurantoin

**DOI:** 10.1007/s00204-023-03572-7

**Published:** 2023-09-04

**Authors:** Sabina Ramirez-Hincapie, Barbara Birk, Philipp Ternes, Varun Giri, Franziska Maria Zickgraf, Volker Haake, Michael Herold, Hennicke Kamp, Peter Driemert, Robert Landsiedel, Elke Richling, Dorothee Funk-Weyer, Bennard van Ravenzwaay

**Affiliations:** 1 BASF SE, Experimental Toxicology and Ecology, Ludwigshafen, Germany; 2 BASF Metabolome Solution GmbH, Berlin, Germany; 3 Pharmacy, Pharmacology and Toxicology, Free University of Berlin, Berlin, Germany; 4 Food Chemistry and Toxicology, Department of Chemistry, RPTU Kaiserslautern-Landau, Kaiserslautern, Germany; 5 Environmental Sciences Consulting, Altrip, Germany

**Keywords:** Metabolomics in vitro, High throughput, Nitrofurantoin, Hepatotoxicity, New approach methodologies, Next generation risk assessment, Point of departure

## Abstract

Omics techniques have been increasingly recognized as promising tools for Next Generation Risk Assessment. Targeted metabolomics offer the advantage of providing readily interpretable mechanistic information about perturbed biological pathways. In this study, a high-throughput LC–MS/MS-based broad targeted metabolomics system was applied to study nitrofurantoin metabolic dynamics over time and concentration and to provide a mechanistic-anchored approach for point of departure (PoD) derivation. Upon nitrofurantoin exposure at five concentrations (7.5 µM, 15 µM, 20 µM, 30 µM and 120 µM) and four time points (3, 6, 24 and 48 h), the intracellular metabolome of HepG2 cells was evaluated. In total, 256 uniquely identified metabolites were measured, annotated, and allocated in 13 different metabolite classes. Principal component analysis (PCA) and univariate statistical analysis showed clear metabolome-based time and concentration effects. Mechanistic information evidenced the differential activation of cellular pathways indicative of early adaptive and hepatotoxic response. At low concentrations, effects were seen mainly in the energy and lipid metabolism, in the mid concentration range, the activation of the antioxidant cellular response was evidenced by increased levels of glutathione (GSH) and metabolites from the de novo GSH synthesis pathway. At the highest concentrations, the depletion of GSH, together with alternations reflective of mitochondrial impairments, were indicative of a hepatotoxic response. Finally, a metabolomics-based PoD was derived by multivariate PCA using the whole set of measured metabolites. This approach allows using the entire dataset and derive PoD that can be mechanistically anchored to established key events. Our results show the suitability of high throughput targeted metabolomics to investigate mechanisms of hepatoxicity and derive point of departures that can be linked to existing adverse outcome pathways and contribute to the development of new ones.

**Supplementary Information:**

The online version contains supplementary material available at 10.1007/s00204-023-03572-7.

## Introduction

The realization of the vision of “toxicity in the twenty-first century” has significantly progressed since the publication of the NRC report in 2007 (National Research Council 2007). Scientific and technical advances of the last decades have fostered the development of numerous cell-based methods, high throughput systems and in silico models as alternative approaches to in vivo animal testing. These new approach methodologies (NAMs) have contributed to the understanding of mechanisms of toxicity and have played an important role in the development of adverse outcome pathways (AOP) (Krewski et al. 2020; Vinken 2013). While the development of these new methods has been instrumental for the evolution of toxicology, the number of chemicals in the market for which there are insufficient toxicological data are evidencing the pressing need to increase the implementation of NAMs in human and environmental risk assessment (Stucki et al. 2022).

Conventional toxicological in vitro testing relies largely on the evaluation of single endpoints, which in most cases is not sufficient for a comprehensive risk assessment and not always translates well to the in vivo situation (Ball et al. 2022; Dix et al. 2007). The implementation of Omics technologies enable the simultaneous measurement of multiple cellular endpoints, providing a multiparametric and comprehensive assessment of different biochemical pathways in a single sample (García-Cañaveras et al. 2016). In particular metabolomics, described as the systematic study of small endogenous molecules known as metabolites, represents the last step in the Omics cascade and as such provides an insight into the current physiological state of an organism including biological responses to external factors such as xenobiotics and therapeutic agents (Guijas et al. 2018). Thus, metabolomics is the “omics” technology that closest represents the phenotype and for this reason has been considered to be closer to classical toxicology than other Omics techniques (Ramirez et al. 2013). Metabolomics approaches have been successfully employed in toxicity assessment for identifying mechanisms of toxicity and characterizing key molecular events (Birk et al. 2021; Cuykx et al. 2018; Kamp et al. 2012; Mattes et al. 2014; Van Ravenzwaay et al. 2007, 2015). For cell-based metabolomics, however, requirements for large biomass quantities have previously restricted the throughput scalability, limiting the testing to few concentrations and single (static) time points (Cuykx et al. 2018; García-Cañaveras et al. 2016; Ramirez et al. 2018). These factors reduce the potential of dose and time-based calculation of dose–response metrics, hampering the applicability of in vitro metabolomics systems in risk assessment (Olesti et al. 2021). In addition, such information may also contribute to discriminate between adaptive and adverse changes.

Given that the liver is one of the main target organs, early mechanistic-based identification of potential hepatotoxins is a highly relevant issue for the pharmaceutical and chemical industry. Recently, in vitro liver models have been employed to derive metabolomics-based points of departure (PoDs) via benchmark concentration modeling (Crizer et al. 2021; Malinowska et al. 2023).

We have previously developed and standardized a high throughput, targeted LC–MS-based in vitro metabolomics platform for the identification and differentiation of liver toxicity Modes of Action (MoAs) in HepG2 cells (Ramirez-Hincapie et al. 2023). Importantly, this assay measures a set of pre-identified metabolites representative of main cellular pathways. Due to its high throughput nature, a broad range of concentrations, covering key points of the dose–response curve can be assessed, offering the possibility of studying substance effect dynamics and allowing accurate and mechanistic anchored metabolome-based PoD estimations.

The aim of this study was to generate metabolome-based dose–response and time series analysis which can be useful to derive dose response metrics from metabolomics data. For this aim, we have selected nitrofurantoin as a model compound. Nitrofurantoin is an antibiotic employed in clinical practice to treat urinary infections. For humans, nitrofurantoin presents a significant drug induced liver injury (DILI) concern, classified as a well-known cause of liver injury (Serrano 2014). The activation of cellular oxidative stress response pathways by nitrofurantoin has been previously demonstrated (Wijaya et al. 2021). At low doses, nitrofurantoin has been shown to activate the endogenous antioxidant machinery by being a potent stimulator of intracellular glutathione synthesis (Wijaya et al. 2022), at high concentrations however, it has been linked to oxidative stress-related hepatotoxicity (Wang et al. 2008). Because of its characteristic dose-related biological responses nitrofurantoin was considered as a suitable compound to assess the applicability of the high throughput targeted metabolomics to provide a basis for a mechanistic-grounded PoD determination.

## Materials and methods

### Cell culture

HepG2 cells (ECACC, UK, maximum passage number 9) were maintained and grown on Dulbecco’s MEM media supplemented with 1 v/v% of penicillin/streptomycin, l-glutamine (200 mM, 1% v/v), non-essential amino acids (1% v/v) and 10% FBS (PAN-Biotech, Aidenbach, Germany) in 75 cm^2^ culture flasks (TPP, Switzerland). For cell passaging (~ 80% confluency) media was removed and cells were washed twice with pre-warmed calcium and magnesium free Dulbecco’s PBS (PAN-Biotech, Aidenbach, Germany). Trypsin was used for cell detachment. A fraction of the cell suspension was then transferred to a new culture vessel. For experiments, 15.000 cells per well (passage 5–9) were seeded in 96-well flat-bottom plates (TPP, Switzerland) and incubated for 24 h for cell attachment (37 °C and 5% CO_2_). After 24 h, culture media were exchanged, and the test substance was added in five concentrations (0.5% DMSO) and incubated in a corresponding 96-well-plate per time point for 3, 6, 24 and 48 h at 37 °C and 5% CO_2_. 72 h post seeding, the assay was stopped by quenching all plates with isopropanol 80% and freezing at − 80 °C. See “Metabolomics Experiments” for more details.

### Test substances

Nitrofurantoin (≥ 98%) and bezafibrate (≥ 98%), used as a positive/quality control in each experiment, were purchased from Sigma Aldrich (Buchs, Switzerland)*.* DMSO (+ 99.8%,) was used as a solvent and vehicle control at a final concentration of 0.5% Thermo Fisher (Geel, Belgium).

### Cytotoxicity and cell viability testing

Commercially available cytotoxicity (CellTox™ Green) and ATP content based (CellTiter-Glo^®^) assays (Promega GmbH, Walldorf, Germany) were multiplexed in a single 96 well-plate following the manufacturer’s instructions. For positive controls, lysis solution 25× was added in wells containing vehicle control treated cells (0.5% DMSO). Fluorescence was measured at *λ*_ex_ = 485–500 nm/*λ*_em_ = 520–530 nm in the GloMax^®^-Multi Detection System (Promega). Luminescence was measured in the GloMax^®^-Multi Detection System (Promega) and was normalized to the values of the vehicle control. Cytotoxicity and ATP cell viability analysis were carried out for range finder pre-tests and in parallel with metabolomics experiments in plates handled and treated exactly as the ones used for metabolite profiling.

### Range finder experiments for concentrations selection

Concentration levels for the metabolomics experiments were based on range finder experiments. Nitrofurantoin was administered to HepG2 cells in 14 concentrations ranging from 0.234 to 1920 µM to following two-fold serial dilutions and incubated for 48 h (6 replicates per concentration). Viability and cytotoxicity tests were performed as described previously. Luminescence values resulting from ATP measurement (CellTiter-Glo^®^ assay) were used to build dose response curves. Curve fitting and effective concentrations (ECs) values were calculated in R using four-parameter Weibull model (W2.4). Calculated EC values were rounded to the nearest integer number for dose selection.

### Live-cell imaging

To monitor cell proliferation, total well confluence was obtained by real time cell imaging analysis using IncuCyte S3 device placed in a normal incubator at 37 °C with 5% CO_2_. Whole-well scans were taken every 1.5 h during the duration of the assay and evaluated using automated phase-contrast analysis (phase mask).

### Metabolomics experiments

**Fig. 1:**
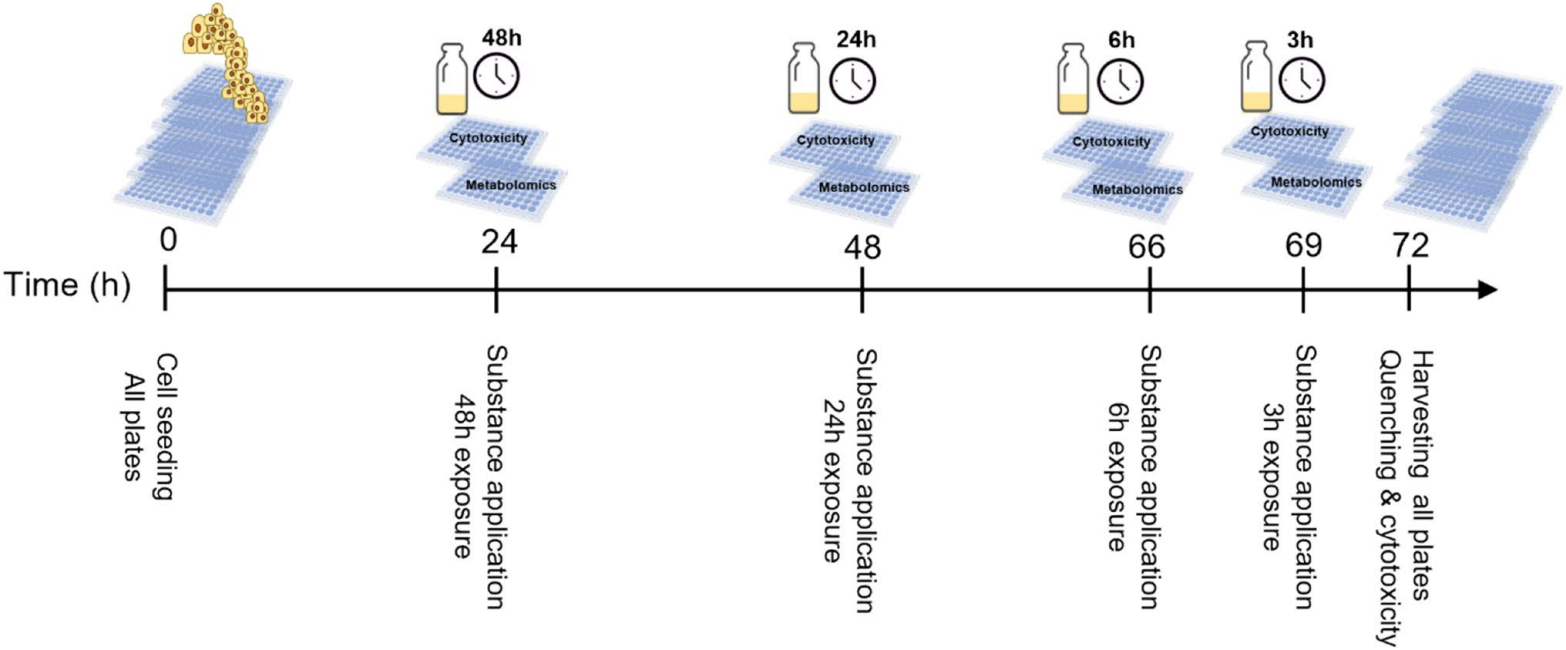
Nitrofurantoin administration for metabolomics experiment. 15,000 HepG2 cells were seeded per well in 96-well plates and incubated for 24 h for initial cell attachment. 24 h post seeding, nitrofurantoin was administered in 5 concentrations (EC_1(ATP)_: 7.5 µM, EC1_15(ATP)_: 15 µM, EC_25(ATP)_: 30 µM, EC_50(ATP)_: 60 µM_,_ EC_85(ATP)_: 120 µM) in a corresponding 96-well-plate per time point by reverse application. After 72 h post seeding, the assays were stopped simultaneously by washing the wells once with 100 µl of 0.9% NaCl followed by snap freezing the plates on liquid nitrogen for 5 s. Metabolomics plates were placed immediately on dry ice and stored at − 80 °C until LC–MS/MS analysis while cytotoxicity plates were used for ATP content and membrane integrity multiplexed assays

After 24 h of cell attachment, substances were administered in 0.5% DMSO (final concentration) to HepG2 cells in five concentrations [EC_1(ATP)_, EC1_15(ATP)_, EC_25(ATP)_ EC_50(ATP),_ EC_85(ATP)_] in a corresponding 96-well-plate per time point. To harvest all plates simultaneously, aiming to achieve same final cell number, treatment was applied by reverse application. (1) 24 h post seeding (48 h substance exposure), (2) 48 h post seeding (24 h substance exposure), (3) 66 h post seeding (6 h substance exposure) and 4) 69 h post seeding (3 h substance exposure) (Fig. 1).

After 72 h post seeding, the assays were stopped simultaneously by first removing the media with a multi-well aspirator and then washing the wells once with 100 µl of 0.9% NaCl followed by snap freezing the plates on liquid nitrogen for 5 s. Plates were placed immediately on dry ice and stored at − 80 °C until LC–MS/ MS analysis.

For each time point, one 96 well-plate was set up with 6 replicates per concentration, 12 replicates for vehicle controls (0.5% DMSO), 6 replicates for positive controls (Bezafibrate 1000 µM) and 6 replicates for blank controls (media without cells). Bezafibrate has been shown to provide a clear and consistent metabolic response (BASF, unpublished results) that is used to confirm the quality of the cell batch used in the analysis. To minimize potential evaporation, the outer rows and columns of the plate were omitted for seeding cells samples and were instead filled with PBS. These plate positions were later on used for technical and linearity checkups during the metabolome analysis (see LC–MS/MS metabolomics section and suppl. Fig. 1 for details on plate set up). Reference samples prepared from lyophilized untreated HepG2 cells were measured in parallel throughout the entire analytical process (QC technical replicates). Data from each metabolite in each sample were normalized against the median of the same metabolite in all reference samples on the same plate to give normalized ratios. Lyophilized HepG2 cells reference samples were used to account for variability between plates (inter- and intra-instrumental variation) and in concentration series (0%, 25%, 50%, 75%, 100%, 125%, 150%, 200%) for linearity checks.

### LC–MS/MS metabolomics

Metabolite profiling of cells was performed directly in the 96-well plate according to a standardized protocol described below.

For quenching and extraction 120 µl of isopropanol 80%, containing internal standards (quality control only, not used for normalization) were added to each well of the frozen samples plate. Afterwards, plates were shaken for 5 min, 750 rpm at 20 °C and placed for 30 s in the ultrasonic device. Then, the plates were centrifugated for 10 min, at 5485 g, 15 °C. 2.5 µl of the extract were injected each for reversed-phase and hydrophilic interaction liquid chromatography followed by MS/MS detection (AB Sciex QTrap 6500+) using the positive and negative ionization mode. For reverse-phase high performance liquid chromatography (RP-HPLC, Ascentis Express C18, 5 cm × 2.1 mm, 2.7 µm. Supelco), gradient elution was performed with mobile phase A, water/methanol/0.1 M ammonium formate (1:1:0.02, w/w), and B, methyl-tert-butylether/2-propanol/methanol/0.1 M ammonium formate/formic acid (4:2:1:0.07:0.035, w/w) (linear gradients, 0 min 100% A, 0.5 min 75% A, 5.9 min 10% A, 600 µl/min). HILIC (ZIC-HILIC, 10 cm × 2.1 mm, 3.5 µm, Merck) gradient elution was performed with mobile phase C, acetonitrile/water (99:1, v/v) with 0.2% (v) acetic acid, and D, 7 mM ammonium acetate with 0.2% (v) acetic acid (linear gradients, 0 min 100% C, 5 min 10% C, 600 µl/min).

Due to the high sample number, the analysis was performed in batches with each batch comprising one 96-well plate. To ensure that the analytical system was suitable for measurement, for each analysis batch a solvent (80% isopropanol) and two external standard calibration samples (covering 213 lipid and polar metabolites) followed by another solvent sample were run at the start of the analysis. The border wells of the 96-well plate were used for linearity samples (resulting in 4 replicates per concentration (except 100% with 5 replicates − the latter samples are used for normalization as described below). This setup ensured that data can be compared across analysis batches. Each 96-well plate was analyzed row-wise starting from well A1. This way one replicate from each treatment was run followed by two linearity samples (in column A and H) before moving to the next treatment replicate.

For bioanalytical quality control, the linearity samples were evaluated regarding coverage (signal in > 80% of linearity samples), linearity (*R*^2^ > 0.64), variability (RSD < 0.6 for the 100% linearity samples) and blank contribution (blank signal < 40% of the 100% linearity samples). When a metabolite failed the quality control check, data for this metabolite were excluded (Ramirez-Hincapie et al. 2023).

### Metabolomics data analysis

To correct for small differences in cell numbers within and between different treatment groups, data were also normalized to the within sample median, as described in detail by Ramirez et al. (2018). For intracellular metabolomic analysis, the median of each sample was calculated across all the 256 measured metabolites.

To generate metabolic profiles for the different concentrations and time points, heteroscedastic *t* test (Welch test) was applied to log-transformed normalized metabolite data to compare treated groups with their respective controls.

To investigate the experimental variability, the variance of every log-transformed metabolite for both pooled samples (technical replicates) and control samples was calculated. These variances were back-transformed to linear scale, yielding a relative standard deviation (RSD) using the following formula:

$$ {\text{RSD }} = { 1 } - { 1}0^{{ - {\text{SDlog}}}} . $$

Principal component analysis (PCA) analyses were performed using R software environment (https://www.r-project.org/) using the ropls package (Thévenot et al. 2015) with log_10_-transformed input data and standard scaling. The input data were normalized to the median of each metabolite in the control samples on each 96-well plate to compensate for differences between plates.

The binomial distribution enrichment analysis was performed using Excel. For this purpose, the number of significant changes (s) at *p* value < 0.05 were counted per treatment and ontology class. The binomial distribution test is used to indicate the probability of a specific number of successes (the number of significant changes) occurring from a specific number of independent evaluations (total metabolites number in the given ontology class). The resulting *p* value for this enrichment is indicated (as category) by color in the tables (grey, light yellow or intense yellow).

## Point of departure derivation

A concentration-dependent response was modeled based on PC1 values obtained from the PCAs. PC1 values for each sample were plotted against the test concentration and a 3-parameter log-logistic model was fitted through the data, using ‘drc’ package (Ritz and Streibig 2005). A confidence interval of 95% was used for the dose–response curve and the control variability was described by the 2.5% and 97.5% quantiles, which correspond to 95% spread of the controls. The PoD marks the concentration at which the confidence interval of the curve surpasses the corresponding quantile of the controls, i.e., the curve with its 95% confidence interval has crossed the 95% spread of the controls.

## Results and discussion

### Range finder pre-test for concentration selection

**Fig. 2:**
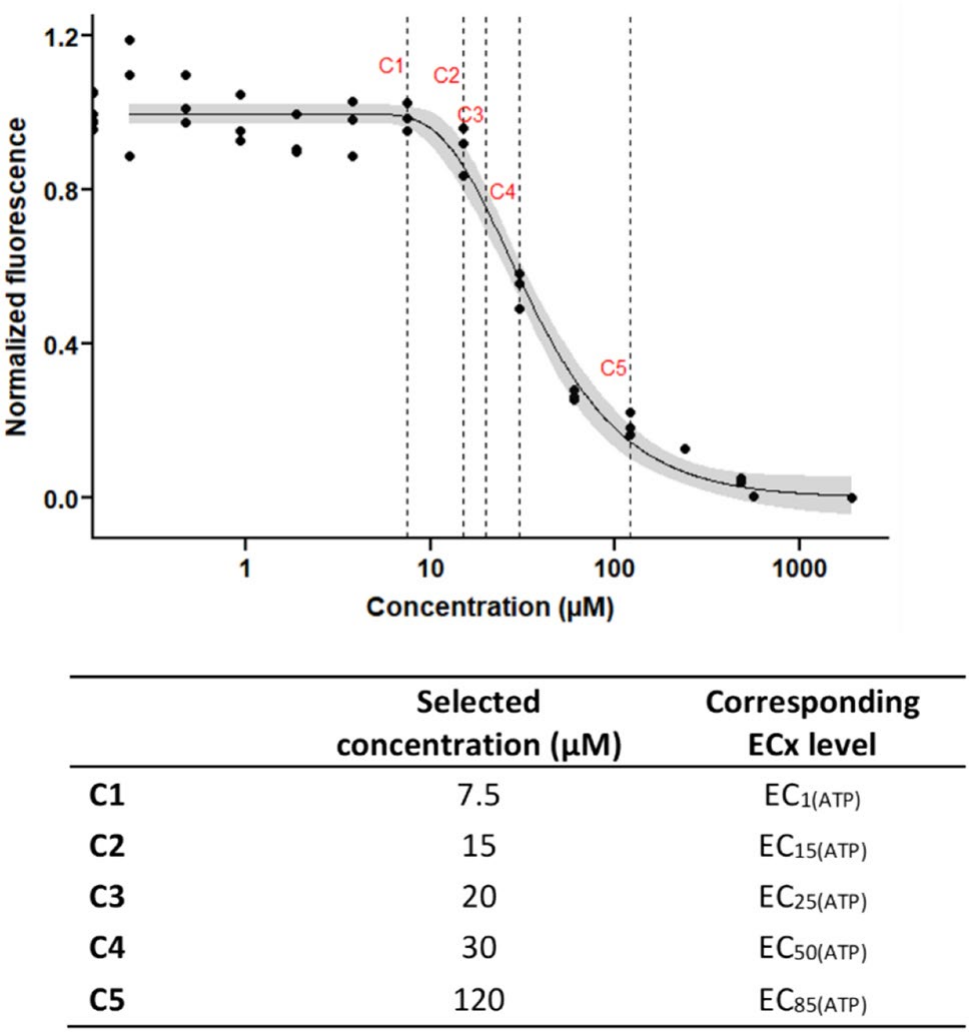
Nitrofurantoin concentration selection for metabolomics experiments. ATP values obtained from the viability pre-test were used to build a dose–response curve. Five concentrations (indicated by the dotted lines) were selected. Upper panel; dose–response curve. Lower panel; corresponding estimated EC concentrations. ECs were estimated based on the computationally fitted ATP dose response curves generated in the range finder experiments upon 48 h of exposure. Five test concentration levels (indicated by the dotted lines) were set based on the dose–response curve generated from ATP measurement (CellTiterGlo^®^) pre-test (Suppl Fig. 2). Values were approximated to the nearest integer number

Initial range-finding experiments were conducted to guide the concentration selection for the metabolomics experiments. After administering increasing concentrations of nitrofurantoin following two-fold serial dilutions (from 0.234 to 1.920 µM), cytotoxicity and cell viability were assessed in parallel upon 48 h of exposure (Suppl Fig. 2). CellToxGreen, a cell impermeable DNA-binding dye which measures membrane integrity was used to identify concentrations that caused overt cell death. ATP production, a more sensitive endpoint expected to reflect earlier alterations in cellular metabolism, was used to generate a dose response curve and derive effective concentration values (EC). Based on ATP content viability pretest, five nitrofurantoin concentrations [C1:EC_1(ATP)_, C2; EC_15 (ATP)_, C3:EC_25(ATP)_, C4:EC_50 (ATP)_, C5:EC_85(ATP)_] were selected for the following metabolome experiment (Fig. 2). The concentration selection aimed to cover important aspects of the concentration response dynamics from no and mild effects to hepatotoxic-related effects. EC_1(ATP)_ was selected to evaluate non-toxic but potentially mild metabolic effects, EC_15(ATP)_ and EC_25(ATP)_ was selected to obtain a moderate substance effect, however, within a low cytotoxicity range and EC_50(ATP)_ and EC_85(ATP)_ were chosen to identify hepatotoxic related metabolite patterns.

### Experimental cytotoxicity and cell viability of selected test concentrations

A critical factor of metabolomics experiments is to distinguish substance-specific effects from unspecific effects produced by overt cytotoxicity. To experimentally assess the effect of the selected test concentrations on both cell viability and cytotoxicity, ATP content (CellTiterGlo) and cell death (CellToxGreen) assays were multiplexed and measured in parallel with the metabolomics experiment in plates handled and treated exactly as the ones used for metabolomics (Suppl. Figure 3).

The estimated ECs calculated in the pre-test from ATP values after 48 h of exposure corresponded closely to the experimentally obtained values in the low [EC_1(ATP)_] and high effect area [EC_50(ATP)_, EC_85(ATP)_] of the dose response curve. In comparison to the vehicle treated cells, the estimated EC_15ATP_ resulted in a mild experimental reduction of ATP (down to 94%) while the EC_25ATP_ caused a higher-than-anticipated reduction in ATP levels (down 63%), suggesting a steep slope in the dose response curve.

At the three highest concentrations [EC_25(ATP)_, EC_50(ATP)_ and EC_85(ATP)_] and the last time point (48 h), ATP levels were markedly affected when compared to untreated controls, suggesting significant impairments in the cellular energy generation. However, this apparent drastic “loss” in viability failed to induce significant cell death measured by means of membrane integrity, suggesting a cytostatic rather than a cytotoxic effect of the highest nitrofurantoin concentrations. Cell growth was monitored by real time imaging during the duration of the assay. After 48 h of exposure, the concentrations corresponding to EC_25(ATP)_, EC_50(ATP)_ and EC_85(ATP)_ had a clear impact on the cellular growth rate (Suppl. Figure 4), confirming the cytostatic effect that resulted in lower cell numbers and consequently produced an apparent reduction in viability when compared to untreated cells. These findings point out that experimental concentration selection based only on viability markers such as ATP, could potentially overestimate the substance cytotoxic effect missing important markers of potential adversity and resulting in an incomplete coverage of the substance response effect. Our results indicate that including additional parameters such as the parallel assessment of two different endpoints in cytotoxicity readouts is important for proper concentration selection and data interpretation.

### Metabolomics experiments

The HepG2 cell line was selected due to its unlimited lifespan, stable phenotype, availability, reproducibility, easy handling, and low cost. Although the limited drug metabolizing and transport capabilities of HepG2 cells are well acknowledged, a comparable stimulation of de novo synthesis of glutathione and gene expression profiles were found in primary human hepatocytes (PHH) and HepG2 when exposed to different nitrofurantoin concentrations (Wijaya et al. 2022) indicating the suitability of the HepG2 cells to investigate nitrofurantoin dynamics.

To study the metabolite dynamics upon nitrofurantoin exposure, HepG2 cells were treated with five different concentrations (C1:7.5 µM, C2:15 µM, C3:30 µM, C4:60 µM, C5:120 µM) at four time points (3, 6, 24, 48 h). A total of 256 unique metabolites was measured of which 181 were annotated and 75 remained unknown. Annotated metabolites were allocated in 13 different metabolite classes such as amino acids, carbohydrates, energy metabolism, nucleobases, vitamins and cofactors and diverse lipid classes (Suppl. Figure 5).

Relative standard deviation (RSD) values of the individual metabolites in the control samples ranged from 8.7% (1st quartile) to 18.7% (3rd quartile) with a median of 13.1% after control normalization (to compensate for differences between plates). The median RSD values of the individual metabolites in the control samples on individual plates ranged from 10.6 to 13.3%. The median RSD values of the individual metabolites in the technical replicates on the individual plates were between 9.2 and 10.3% (Suppl Fig. 6). The experimental variability of the technical (QC samples) and biological (vehicle) controls in our study was thus well below the recommended threshold of 30% (Viant et al. 2019).

### Metabolome analysis of nitrofurantoin-treated cells shows concentration and time response effects

**Fig. 3:**
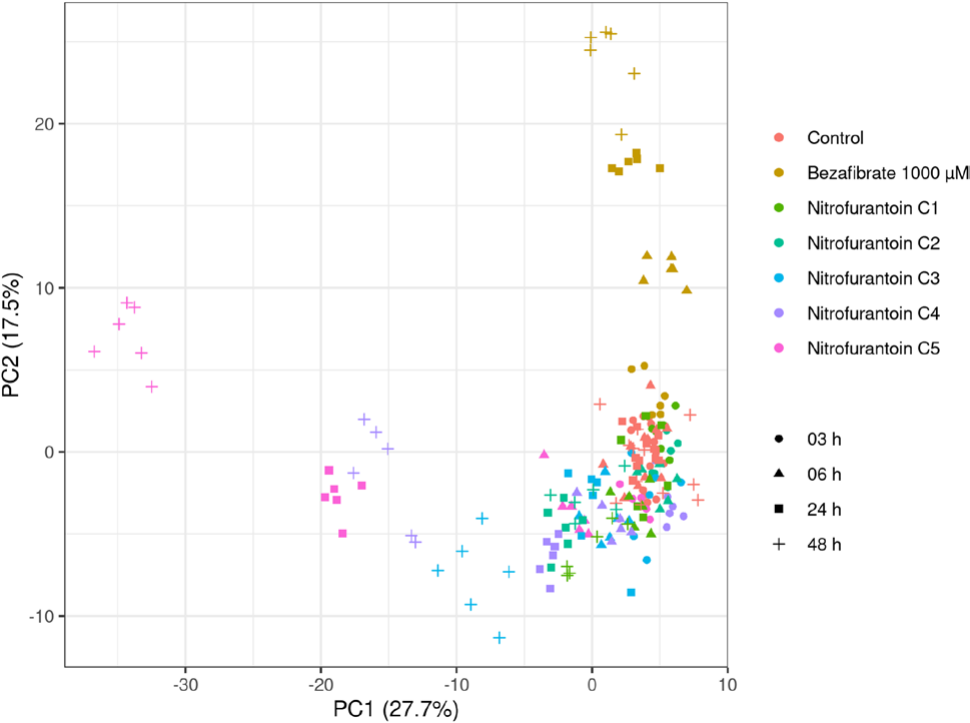
PCA of nitrofurantoin metabolic profiles show time and concentration response effects. PCA analysis of the metabolic profiles of HepG2 cells upon nitrofurantoin treatment. Bezafibrate was used as a positive control. C1:7.5 µM, C2:15 µM, C3:30 µM, C4:60 µM, C5:120 µM

**Fig. 4:**
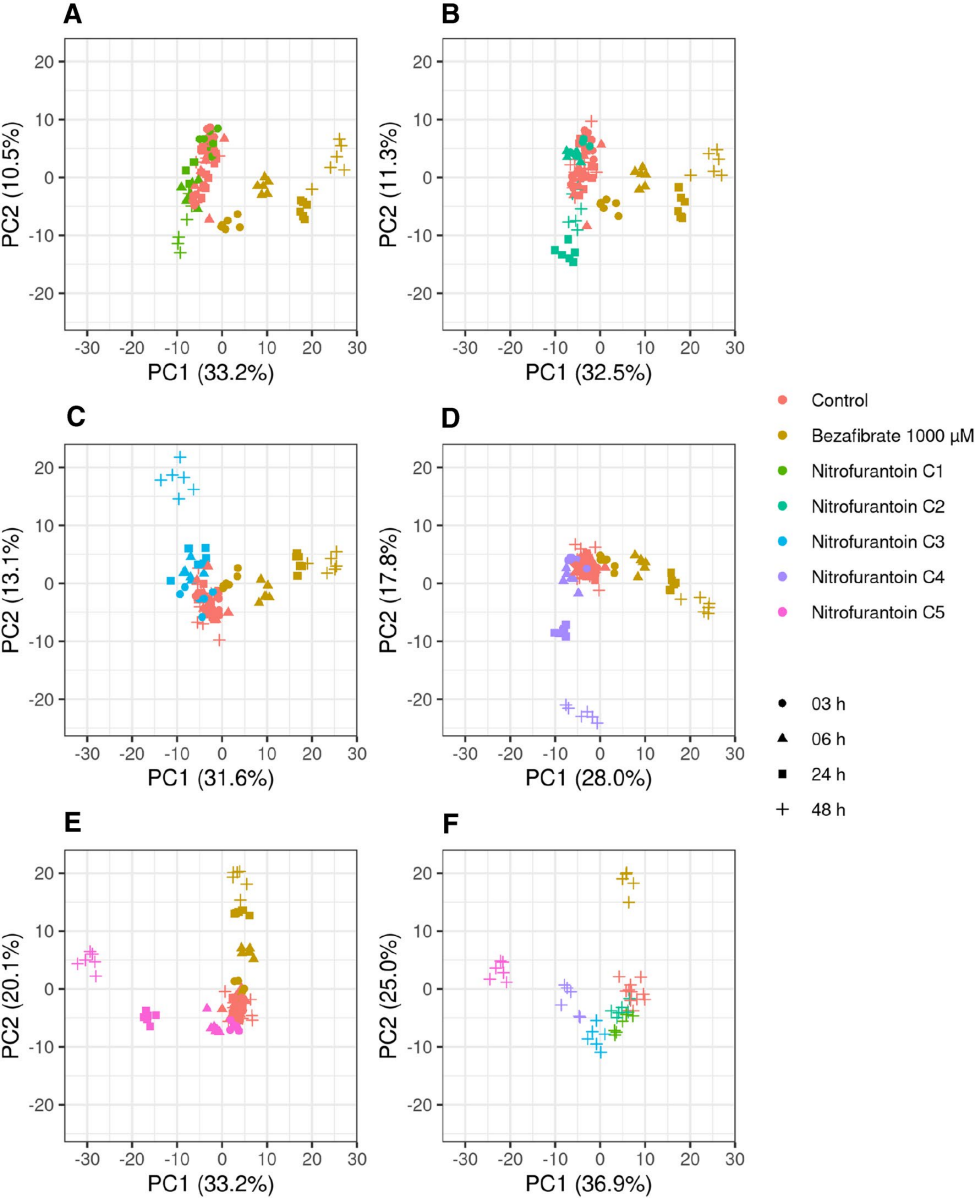
PCAs of metabolomics time-response effect for each tested concentration. **a** C1: 7.5 µM, **b** C2: 15 µM, **c** C3: 30 µM, **d** C4: 60 µM, C5: 120 µM and **f** metabolic profiles of the five tested concentrations: C1–C2, upon 48 h of exposure

Metabolite profiles of cells treated with five difference concentrations of nitrofurantoin (C1–C5) for 3 h, 6 h, 24 h or 48 h were first analyzed by PCA (Fig. 3). Both concentration- and time-dependent responses to the nitrofurantoin treatment were observed. To minimize time-related effects that are not connected to the nitrofurantoin exposure, all cells were quenched at the same time point (72 h after seeding), and nitrofurantoin was applied at a respective earlier time point (69 h, 66 h, 48 h, and 24 h after seeding). After 3 h of exposure, none of the tested concentrations induced a visible effect. At the lowest tested concentration (C1), significant effects were observed only after 48 h of exposure (Fig. 4A). From the C2 onwards, clear treatment effects were evident already after 24 h of exposure (Fig. 4B–D). The strongest treatment effect and highest resolution of concentration and time effects was observed at the highest concentration (C5) and latest time point (48 h) (Fig. 4E, F). Our findings suggest that at low concentrations, nitrofurantoin exposure times below 6 h are not sufficient to produce an identifiable effect on the metabolome, while at higher concentrations, effects can already be identified after shorter exposure times. In line with our observations, Malinowska and coworkers evaluated the variability of the HepaRG cellular baseline metabolome at different time points, suggesting that a reliable detection of metabolic changes upon a toxicant exposure is achieved after a minimum exposure time of 6 h (Malinowska et al. 2022), which is largely in line without our observations. It is conceivable that the susceptibility of HepG2 cells changes with the degree of confluency of the culture (time after seeding). It is formally possible that the effects of shorter nitrofurantoin exposure times on the metabolome are smaller just because nitrofurantoin was applied at a higher confluency (later time after seeding), when the cells were less metabolically active and potentially less susceptible to nitrofurantoin. Investigating such interactions would require a different experimental setup than in the present study, which was designed to minimize time-related effects on the metabolome (irrespective of treatment) by quenching all samples at the same time point after seeding. We consider it unlikely, however, that the marginal difference in physiological age would have had a significant influence on the nitrofurantoin mediated chemical effect. In a previous paper, in which we reported on the development of this metabolomics in vitro system, we demonstrated that even different cell passage numbers had no effect on base line metabolomics (Ramirez-Hincapie et al. 2023).

Following the PCA evaluation, metabolic profiles of nitrofurantoin-treated cells were subjected to univariate statistics to identify changes in individual metabolites. A univariate enrichment analysis was carried out to evaluate the number of significantly changed metabolites per ontology class (Suppl. Figure 7). The data revealed dose and time dependency, with increasing number of altered metabolites at higher concentrations and later time points. These results demonstrate that the high throughput in vitro metabolomics assay presented here is able to distinguish the effects at different concentrations and time points and therefore is suitable to perform metabolome-based time and dose responses analysis.

The implementation of tools such as the one presented here allows to integrate a temporal dimension in the assessment of compound metabolic dynamics. This type of information not only provides mechanistic temporal insights but is also valuable for the selection of relevant in vitro sampling time points for risk assessment.

### Metabolite dynamics over time and concentrations show differential profiles as potential indicators of initial, adaptive, and toxic responses

Heatmaps of metabolite changes per class were generated in order to assess the metabolic dynamics over concentration and time (Suppl Fig. 8). This type of analysis allows the identification of key metabolites or metabolite class dynamics useful to follow up on the development and progression of a hepatotoxic phenotype. Suppl Fig. 8A shows metabolite changes by class over the different exposure times while Suppl Fig. 8B depicts the metabolite changes per class as a function of the applied compound concentration.

Predicting adversity from omics data remains an important limitation for the use of these technologies in risk assessment (Olesti et al. 2021). Therefore, the investigation of multiple endpoints at various time-points is fundamental to understand the progression of different key events along an adverse outcome pathway (AOP). By evaluating consistent metabolite changes in low, medium, and high concentrations at different time points, we generated a dataset which closely captured the previously reported nitrofurantoin effect evolution and allowed to identify differential pathway activation and metabolic markers potentially indicative of a transition from adaptive to adverse effects (Table. 1).

**Table 1:**
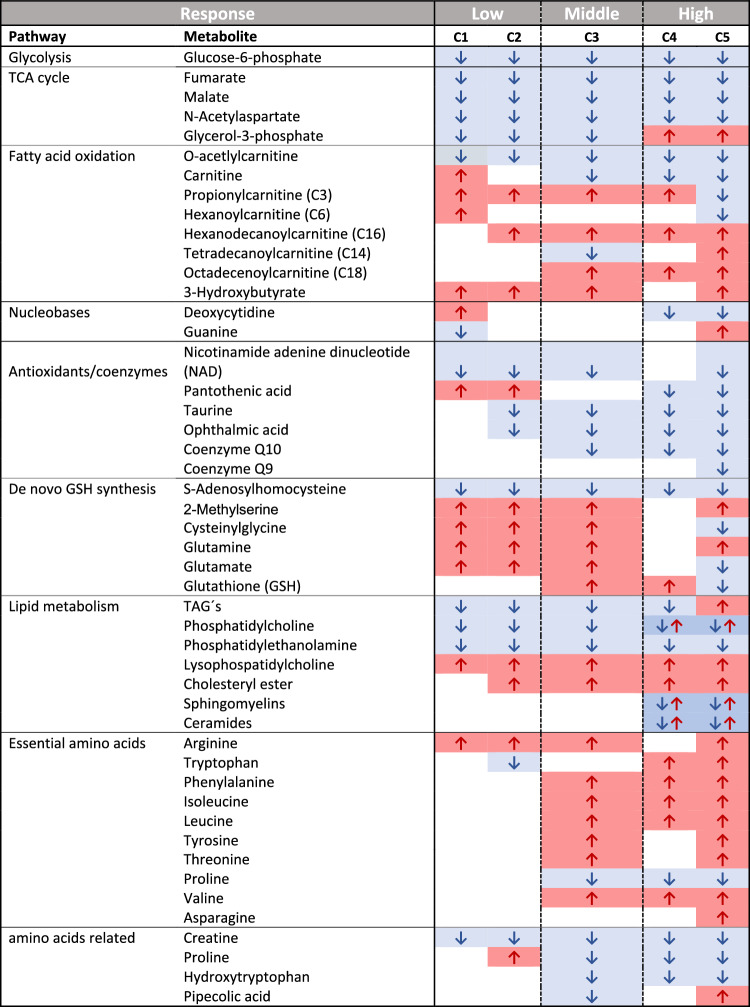
Characteristic metabolite changes of early, adaptive and hepatotoxic nitrofurantoin response

Consistent metabolite changes in metabolic profiles of HepG2 cells treated with low (C1: 7.5 µM, C2: 15 µM) middle (C3: 30 µM) and high (C4: 60 µM, C5: 120 µM) nitrofurantoin concentrations. Red arrows represent elevated levels and blue arrows represent reduced levels. Changes are calculated relative to the controls. Consistent time response changes are depicted; the majority of consistent changes were evident upon 24 h of exposure. In the the highest concentrations, some consistent changes were evident already upon 6 h of exposure

#### Metabolic profile of cells exposed to low concentrations

Low concentrations (C1 and C2), corresponding to 7.5 µM and 15 µM, showed no significant reduction in viability, as well as no cell death or detrimental effects on the cell growth. Metabolic profiles of cells treated with these concentrations exhibited alterations mainly in the energy and lipid metabolism as well as in antioxidant molecules as an early response to nitrofurantoin exposure. Concentrations of TCA cycle metabolites (fumarate and malate), glycolysis intermediates (glucose-6-phosphate) and acetyl-CoA donors (*N*-acetyl aspartate), decreased consistently from the lowest concentrations onwards in a time and dose response manner. Pantothenic acid, a precursor to CoA and a part of the anchoring system of the fatty acid synthase complex, increased in the lowest concentrations and shifted to an increase in the highest (C4–C5). Levels of glycerol-3-phosphate decreased in the lower and middle concentrations (C1–C3) but increased in the highest concentration. Changes in metabolites from the fatty acid oxidation pathway were as well observed in the low concentrations.

*O*-Acetlylcarnitine, the acetylated derivative of carnitine, which facilitates the movement of acetyl-CoA into the mitochondrial matrix during fatty acid oxidation was reduced from the C1 onwards while levels of short and long chain acylcarnitines (propionyl carnitine and hexadecanoyl carnitine), increased consistently from C1–C2 to C4. The observed decreased levels of glycolysis and TCA intermediates together with increased concentrations of acylcarnitines and the ketone 3-hydroxybutyrate, suggest a shift towards β-oxidation for energy production.

Decreased concentrations of the cofactor nicotinamide adenine dinucleotide (NAD), the amino acid taurine and the glutathione analogue ophthalmic acid, indicated the presence of reactive oxygen species (ROS). Through its action as an antioxidant, taurine has been shown to play a role in counterbalancing oxidative stress attenuating the development of liver steatosis in vitro and in vivo (Murakami et al. 2018). In line with our observations of decreased antioxidant molecules, it has been demonstrated that the redox cycling during nitrofurantoin metabolization generates different ROS such as superoxide anion, hydrogen peroxide, and hydroxyl radicals (Wang et al. 2008). In addition, metabolic profiles of cells treated with the lowest nitrofurantoin concentrations, showed that precursors of glutathione such as glutamine, glutamate, cysteinylglycine and 2-methylserine started to increase while S-adenosylhomocysteine decreased possibly as an early indicator of the stimulation of de novo glutathione synthesis as it became evident in the middle concentration (C3).

Some of the earliest metabolic changes with respect to both concentration and time were observed in the lipid metabolism. Decreased levels of triacylglycerols (TAGs) were evident already in the lowest concentration (C1) and from an early time point (6 h onwards). Phosphatidylcholine levels were found reduced across the low and mid doses (C1–C3) while lysophosphatidylcholine concentrations increased in all C1–C5 concentrations. Phosphatidylethanolamine levels decreased in all five tested concentrations while cholesteryl esters showed increased levels from the D2 onwards. Our data evidence that nitrofurantoin exerts significant effects on different lipids species even at low concentrations. These types of alterations in the lipid metabolism have, so far, not been reported in the literature as a direct consequence of nitrofurantoin exposure. These findings add to the current knowledge of nitrofurantoin mechanisms and represent an avenue for future research.

#### Metabolic profile of cells exposed to the middle concentrations

The mid concentration (C3), corresponding to 20 µM, caused a moderate effect on ATP production and cell growth but failed to induce significant cell death. At this concentration, differential changes mainly in metabolites involved in the cellular antioxidant response, the de novo glutathione synthesis and amino acids were observed. Levels of coenzyme Q10 decreased in a concentration and time response manner starting from the middle concentration while glutathione (GSH) increased consistently after 24 h of exposure to the C3. Following the observed GSH dynamics, levels of proline (amino acid synthesized from glutamate) started to decrease from the C3 consistently. Metabolite changes in the middle nitrofurantoin concentration are reflective of a higher utilization of antioxidant molecules and are in line with the reported stimulation of intracellular GSH synthesis by nitrofurantoin (Wijaya et al. 2022).

Levels of essential amino acids (phenylalanine, isoleucine, leucine, tyrosine, threonine, and valine) were significantly increased starting at the C3 concentration onwards resulting in high levels in the highest concentrations. High intracellular levels of amino acids suggest a reduced amino acid utilization. Reduction of protein synthesis has been reported as a common consequence of stress response pathway activation, resulting in increased intracellular amino acids concentrations and reduced cell growth to conserve amino acids and energy and decrease the cellular protein load as one adaptive measure to overcome stresses (Santiago-Díaz et al. 2023). Attenuation of protein translation is characteristic of the unfolded protein response (UPR) pathway activation. UPR-activating compounds are mostly classified as the severe DILI compounds. Nitrofurantoin has been shown to significantly active the UPR pathway in HepG2 cells (Wijaya et al. 2021). Importantly, it has been proposed that UPR response could represent a key predictor for adverse cellular outcomes for DILI compounds (Wijaya et al. 2021).

Through the activation of adaptive cellular stress response pathways, oxidative stress, and endoplasmic reticulum stress (resulting in unfolded proteins) are typically counteracted. Reactive metabolites generated from nitrofurantoin metabolization can be inactivated by the cellular antioxidant defense system (e.g., GSH). The UPR pathway responds to an accumulation of misfolded proteins in the endoplasmic reticulum by restoring the normal function via decreasing protein translation, degrading misfolded proteins, and activating the signaling pathways that lead to increasing the synthesis of molecular chaperones involved in protein folding (Hetz and Papa 2018). Thus, the metabolic profiles at this concentration are potentially reflective of an adaptive phenotype.

#### Metabolic profile of cells exposed to high concentrations

The highest tested concentrations, particularly C5 corresponding to 120 µM, showed significant impairments on cell growth and ATP production which correlated with the strong effect observed in the metabolome. Therefore, metabolites that were differentially altered in the profiles of cells treated with the two highest nitrofurantoin concentrations (C4, C5) were used to identify hepatotoxic responses. GSH concentrations were significantly reduced upon 48 h of exposure to the highest concentration (C5). Alongside GSH reduction, levels of its precursors glutamate cysteinyl glycine and S-adenosylhomocysteine decreased. An excess of reactive metabolites, beyond homeostasis, can modify cellular macromolecules leading to cellular dysfunction. Intracellular levels of antioxidants have been suggested as important regulators of nitrofurantoin-induced cytotoxicity which has been correlated to hepatitis and tissue necrosis observations in vivo (Wang et al. 2008). Particularly, GSH plays an important role in nitrofurantoin detoxification; nitrofurantoin metabolites have been shown to produce a dose-dependent depletion of total cellular glutathione content, likely due to conjugation of drug metabolites with GSH (Spielberg and Gordon 1981). Our results indicate that at the highest concentration, the capacity of cells to synthesize GSH was compromised, leading to the final depletion of GSH rendering the cells vulnerable for ROS damage.

Glycerol-3-phosphate levels increased in the two highest concentrations. This metabolite is involved in transporting reducing equivalents across the mitochondrial membrane via the glycerol phosphate shuttle for oxidative phosphorylation (Liu et al. 2021). Metabolites from the fatty acid oxidation pathway were also changed in the highest concentrations.

Long chain acylcarnitines (tetradecanoylcarnitine) switched from reduced levels in in the low and mid concentrations, to an upregulation at C5. Propionylcarnitine switched from a consistent increase in the lower concentrations to an increase in the highest concentration while tetradecanoylcarnitine changed from decreased levels in the C3 to increased levels at the C5. Alterations in octadecenoylcarnitine (increase) and hexanoylcarnitine (decrease) were uniquely observed at the highest concentrations. Noteworthy, concentrations of TAGs switched from consistently lower levels in C1–C4 to highly increased concentrations in C5.

The high concentrations of glycerol-3-phosphate, long chain acylcarnitines and TAGs together with lower levels of short chain acylcarnitines are reflective of an impairment of the mitochondrial activity and fatty acid β-oxidation pathway. In agreement with our findings, it has been shown that cell viability decreases significantly at nitrofurantoin concentrations higher than 100 μM, accompanied by impaired mitochondrial respiration (Wijaya et al. 2022). In our study, an inhibition of β-oxidation in the highest nitrofurantoin concentrations is evidenced by reduced free carnitine, and an increase in the fatty acid pool. Free fatty acids can incorporate in lipid species such as TAG and ceramides. In our study, the concentrations of both lipid species were highly elevated. Higher levels of TAGs and ceramides are typical findings in liver toxicity studies (Beyoglu and Idle 2013). Accumulation of TAGs is the hallmark of steatosis while high ceramides levels have been implicated in the impairment of different metabolic processes, being considered as lipotoxic species (Kawano and Cohen 2013; Kurz et al. 2019).

Finally, levels of pipecolic acid were increased only at the highest concentration and latest time point (48 h). Significantly elevated levels of pipecolic acid have been found in plasma of patients with chronic liver disease (Fujita et al. 1999).

In summary, the observed metabolomics alterations matched thoroughly with the nitrofurantoin toxicological mechanisms described in literature such as the de novo stimulation of GSH synthesis and the activation of oxidative stress and unfolded protein response pathways in low and middle concentrations, and the mitochondrial impairment and GSH depletion in the high concentrations. Metabolic profiles of cells exposed to low concentrations (C1, C2) revealed initial responses in metabolite changes upon nitrofurantoin exposure. The middle concentration (C3) reflected changes potentially indicative of an adaptive phenotype which progressed into a more severe hepatotoxic metabolic phenotype in the highest concentrations (C4, C5).

### Point of departure determination based on metabolomics data

**Fig. 5:**
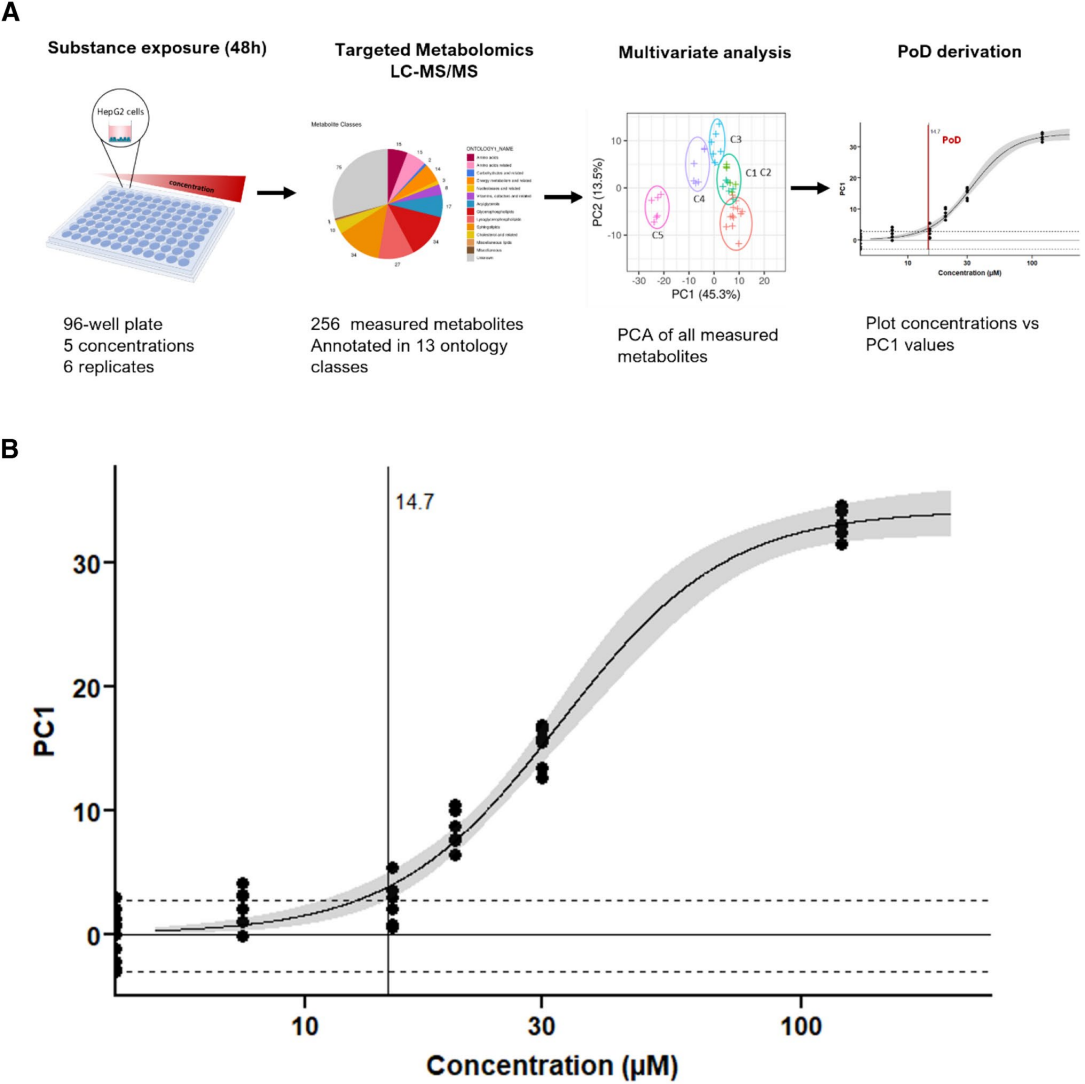
Point of departure (PoD) derivation from metabolomics data. **a** PoD derivation workflow, **b** nitrofurantoin PoD. Global metabolite changes as estimated by principal component analysis (PCA) exhibit exposure concentration dependency. For the PoD derivation, a concentration-dependent response was fitted based on PC1 values obtained from the PCAs of 48 h nitrofurantoin treated cells at five concentrations. PC1 values for each sample were plotted against the test concentration and a 3-parameter log-logistic model was fitted through the data. A confidence interval of 95% was used for the dose–response curve (denoted by the grey ribbon). The spread of controls is marked by the horizontal dashed lines, which represent the 2.5% and 97.5% quantiles; the mean is represented as horizontal solid line. The point of departure (PoD), marked by a vertical solid line, marks the concentration at which the confidence interval of the curve surpasses the corresponding quantile of the controls for the first time

The establishment of human health reference values is a key outcome of chemical risk assessment. For in vitro data, the starting point for the determination of such values includes the derivation of a point of departure (POD) from dose–response modelling followed by an In Vitro–In Vivo Extrapolation (IVIVE) analysis to link an in vitro effect concentration with its in vivo counterpart. The successful application of IVIVE to transform in vitro concentrations into doses expressed in mg/kg bw, as derived in in vivo studies has been demonstrated in various publications (Abdullah et al. 2016; Louisse et al. 2017; Ning et al. 2019; Shi et al. 2020). This approach was also proposed recently by Ball et al., in which a more generalised framework for the transition from in vivo to NAM-based approaches was presented (Ball et al. 2022). Due to their multiparametric nature, Omics technologies allow to measure multiple endpoints and pathways simultaneously, representing a more informative alternative than traditional in vitro studies. Here we explore a PCA-based approach using the complete set of previously annotated metabolites to derive a PoD at each of the different tested time points (Suppl. Figure 9). As the PC1 accounts for the strongest response in metabolome changes for the different test concentrations, PC1 values for each replicate were plotted against the concentration tested. Then, a concentration response curve was fitted and the 95% confidence interval for the curve was determined represented by the grey ribbon (Suppl. Figure 9, Fig. 5). To account for the variability of the controls, the 2.5% and 97.5% quantiles were used, covering 95% of the data (dotted line in Suppl. Figure 9, Fig. 5). The PoD was defined as the point where the 95% confidence interval of the curve diverges from the corresponding quantile of the controls for the first time. The PoD represented the onset of a global change in the metabolome. Above this concentration, early changes in the novo GSH synthesis pathway, energy and lipid metabolism become evident which increased at higher concentrations. The 48 h’ time point showed the most pronounced concentration response resolution, and such it could be presumed that is the most conservative. After exploring the PoD estimations with the different time points, it was observed that, numerically, the PoD from 24 and 48 h is very similar (24 h: 13.7 µM, 48 h: 14.7). The 48 h’ time point showed the most pronounced concentration response resolution; the doses C1–C4 are further apart in the vertical direction (PC1), and therefore, it was assumed to be the most conservative. After exploring the PoD estimations based on the different time points, it was observed that, numerically, the PoD from 24 and 48 h is very similar (24 h: 13.7 µM, 48 h: 14.7 µM). This observation is likely related to the shape of the dose response curve and the data variability, which ended up causing a very similar PoD. However, since the strongest PC1 response was observed after 48 h (D4 and D5 higher in the 48 h than in the 24 h time point), and this time point exhibits a better dose response curve fit and less data variability compared to the 24 h timepoint. If the experiment were to be repeated, 48 h exposure time may give a slightly more conservative PoD estimate since the doses C1–C4 are further apart in the vertical direction (PC1) and will therefore, give a better resolution of the concentration response. Thus, our recommendation is to use the 48 h exposure time for further risk assessment (Fig. 5).

Recent studies have used untargeted metabolomics data to derive PoD via benchmark dose (BMD) (Crizer et al. 2021; Malinowska et al. 2023). These approaches have been based on BMD calculation for single features and lack comprehensive metabolite annotations which could hamper data interpretation. Here we propose an alternative way on how to derive mechanistic-anchored PoD based on the complete set of biological data obtained from metabolomics experiments. Both methods provide a biologically based starting point that can be used to transform the PoD concentration by means of IVIVE into a reference value in expressed in mg/kg bw for human health risk assessment. The advantage of using a broad targeted approach with annotated metabolites is that only with this knowledge adverse outcome pathways can be identified and that an attempt can be made to discriminate between non adverse (adaptive) responses and adverse effects. Differentiation between adverse effects and adaptive responses are a critical consideration for the broad implementation of NAMs and in particular for multiparametric Omics data. Recently, ECETOC has published a paper on their workshop about Omics threshold on non-adversity with particular emphasis on the determination of PoD (Gant et al. 2023).

Adverse responses are considered changes that likely result in impairments of functional capacity, impairments of the capacity to compensate for additional stress or increase the susceptibility to other influences (Keller et al. 2012). Although it is not the purpose of this paper to derive such a value for nitrofurantoin, we believe that using a PC1 approach, takes into account the multiparameter nature of Omics data and is considered more robust than single parameter data. However, further research is needed to identify (groups of) metabolites that are representative of an adverse effect, so that these can be used to derive a PoD for adverse effects and to extrapolate this value into relevant in vivo concentrations for risk assessment.

Recovery studies, for example, can be introduced to further characterize adversity in in vitro studies. It is acknowledged that approaches solely based on biological responses will be conservative, and as such will not underestimate the characterization of hazard and can be used in a tiered approach.

## Conclusion

Recent investigations have shown the potential of applying high throughput untargeted metabolomics approaches to derive hepatotoxicity-related PoD. However, lack of metabolite identification, a characteristic of untargeted methods, challenges the biological interpretations of the results hampering the assessment of the relevance and applicability of these data in safety assessment. In the present study, we have implemented a high-throughput targeted metabolomics platform (covering metabolites from relevant biological pathways) and showed the suitability of the system to elucidate metabolic dynamics over time and concentration to provide a mechanistic-anchored approach to derive and interpret dose and time response metrics from metabolomics data. Both PCA and univariate analysis evidenced clear metabolome-based time and concentration response effects. Mechanistic information allowed to track the differential activation of cellular pathways indicative of early adaptive and hepatotoxic response. At low concentrations, effects were seen mainly in the energy and lipid metabolism, in the mid concentration the activation of the antioxidant cellular response was evidenced by increased levels of GSH and metabolites from the de novo GSH synthesis pathway. At the highest concentrations, the depletion of GSH, accumulation of essential amino acids, ceramides and pipecolic acid together with alternations reflective of mitochondrial impairments, were indicative of a hepatotoxic response. Our results were in line with the broad range of reported concentration-dependent effects of nitrofurantoin. In addition, effects of nitrofurantoin exposure on the lipid metabolism, which to our knowledge have not yet been documented in the literature, were observed. After confirming the mechanistic relevance of the data, we proposed an alternative way to derive metabolomics-based PoD by PCA using the whole set of measured metabolite profiles at each concentration. This approach allows to obtain values from the entire dataset and to derive PoDs that can be mechanistically anchored to established key events. This study demonstrates a very good sensitivity of the high throughput in vitro metabolomics method to explore mechanisms of hepatoxicity, and dynamics progression to potential adversity. However, further studies are needed to define solid parameters for adversity in vitro. Importantly, our work proposes a workflow for PoD derivation that offers the possibility of obtaining mechanistic information and therefore serves to build trust in implementing metabolomics data in risk assessment. Follow up investigations on the integration of these data into in vitro to in vivo extrapolations models (IVIVE) and on the characterization of adaptive vs adverse responses are granted. In the absence of clear guidance to discriminate between adaptive/non-adverse changes and adverse effects, using initial biological responses is a conservative approach which can be used in a tiered system.

This method can be extended to further cell lines and iPSCs for the investigation of different organ toxicities and is suitable for a wide range of next generation risk assessment applications such as MoA investigation, read across and PoD derivation that demand rapid, cost effective and multiparametric high throughput analysis.

## Supplementary Information

Below is the link to the electronic supplementary material.

Supplementary file1 (DOCX 1127 KB)

## Data Availability

The datasets generated during and/or analysed during the current study are available from the corresponding author on request.
