# Supplementary material for: Application of high throughput in vitro metabolomics for hepatotoxicity mode of action characterization and mechanistic-anchored point of departure derivation: a case study with nitrofurantoin
Source: Arch Toxicol. 2023 Sep 4;97(11):2903–17. doi: 10.1007/s00204-023-03572-7 (PMC10504224; doi:10.1007/s00204-023-03572-7)
Supplement: Supplementary file 1 — Supplementary file1 (DOCX 1127 KB) [file 204_2023_3572_MOESM1_ESM.docx]

**Application of High Throughput In *vitro* Metabolomics for Hepatotoxicity Mode of Action characterization and Mechanistic-anchored Point of Departure Derivation: A Case Study with Nitrofurantoin.**

Sabina Ramirez-Hincapie^1^, Barbara Birk^1^, Philipp Ternes ^2^, Varun Giri^1^, Franziska Maria Zickgraf^1^, Volker Haake^2^, Michael Herold^2^, Hennicke Kamp^2^, Peter Driemert^2^, Robert Landsiedel^1,3^, Elke Richling^4^, Dorothee Funk-Weyer^1^, Bennard van Ravenzwaay^5^

^1^ BASF SE, Experimental Toxicology and Ecology, Ludwigshafen, Germany

^2^ BASF Metabolome Solution GmbH, Berlin, Germany

^3^ Free University of Berlin, Pharmacy, Pharmacology and Toxicology, Berlin, Germany.

^4^ Food Chemistry and Toxicology, Department of Chemistry, RPTU Kaiserslautern-Landau, Kaiserslautern, Germany

^5^ Environmental Sciences Consulting, Altrip, Germany

**Supplementary information**

**Suppl Fig. 1 96-well plate set-up.** For each time point, one 96 well-plate was set up with 6 replicates per concentration (C1-C5), 12 replicates for vehicle controls (0.5% DMSO), 6 replicates for positive controls (Bezafibrate 1000µM) and 6 replicates for blank controls (media without cells). To minimize potential evaporation, the outer rows and columns of the plate were omitted for seeding cells samples and were instead filled with PBS. These plate positions were later on used for technical and linearity checkups during the metabolome analysis as follows; dark gray: technical QC samples, not used for final data analysis**.** S: Solvent blank (80% isopropanol)**,** KP: calibration sample containing a mix of polar compounds**,** KL: calibration sample containing a mix of lipid compounds. Light gray: samples used for data normalization and to determine the analytical quality of the individual metabolites as described in Materials & Methods. Pool: reference samples prepared from lyophilized untreated HepG2 cells, used for data normalization. U25–U200: dilution series of the same material as Pool samples, ranging from 25% to 200% (Pool samples correspond to 100%)**,** BL: process blank (corresponds to 0%)**.** Colored background: cell culture experiment**.** Media: culture media without cells, used to check if any remaining culture media after 1× washing with PBS contribute to metabolite levels**,** C1–C5: cells treated with different concentrations of nitrofurantoin**,** Beza: cells treated with bezafibrate, Control: cells treated with vehicle only (0.5% DMSO), Control+1–Control+5: control samples spiked with nitrofurantoin, bezafibrate, and glutathione (reduced and oxidized) *after* quenching, not used in the analysis presented here.


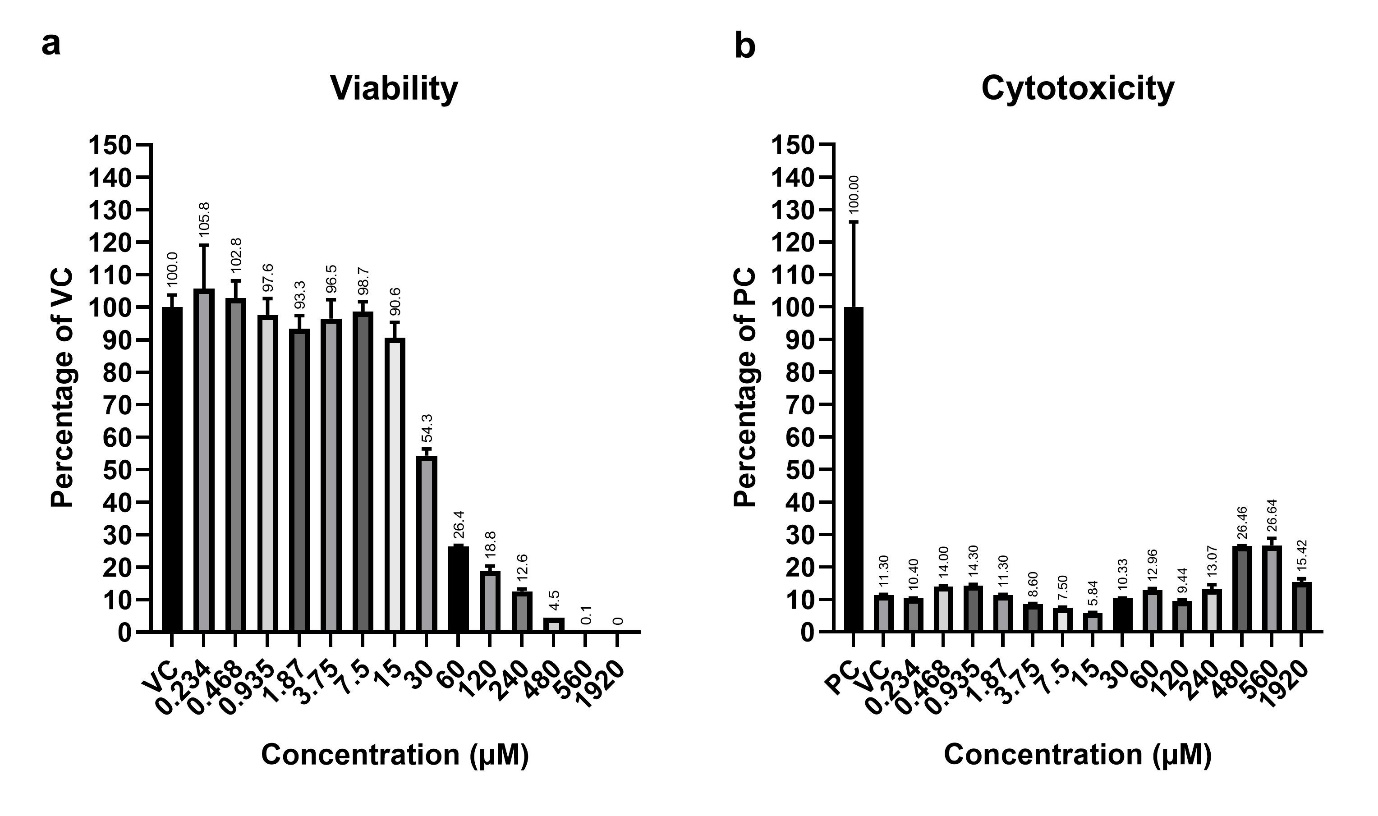


**Suppl Fig.2** **Cytotoxicity and cell viability range finder for dose selection.** (a) Cell viability ATP content-based assay (CellTiter-Glo®) and (b) membrane impermeable DNA-binding dye-based cytotoxicity assay (CellTox™ Green) n=6. Values are presented as percentage of vehicle controls (VC) for CellTiter-Glo® and as percentage of positive control (PC) (lysis buffer) for CellTox™ Green.


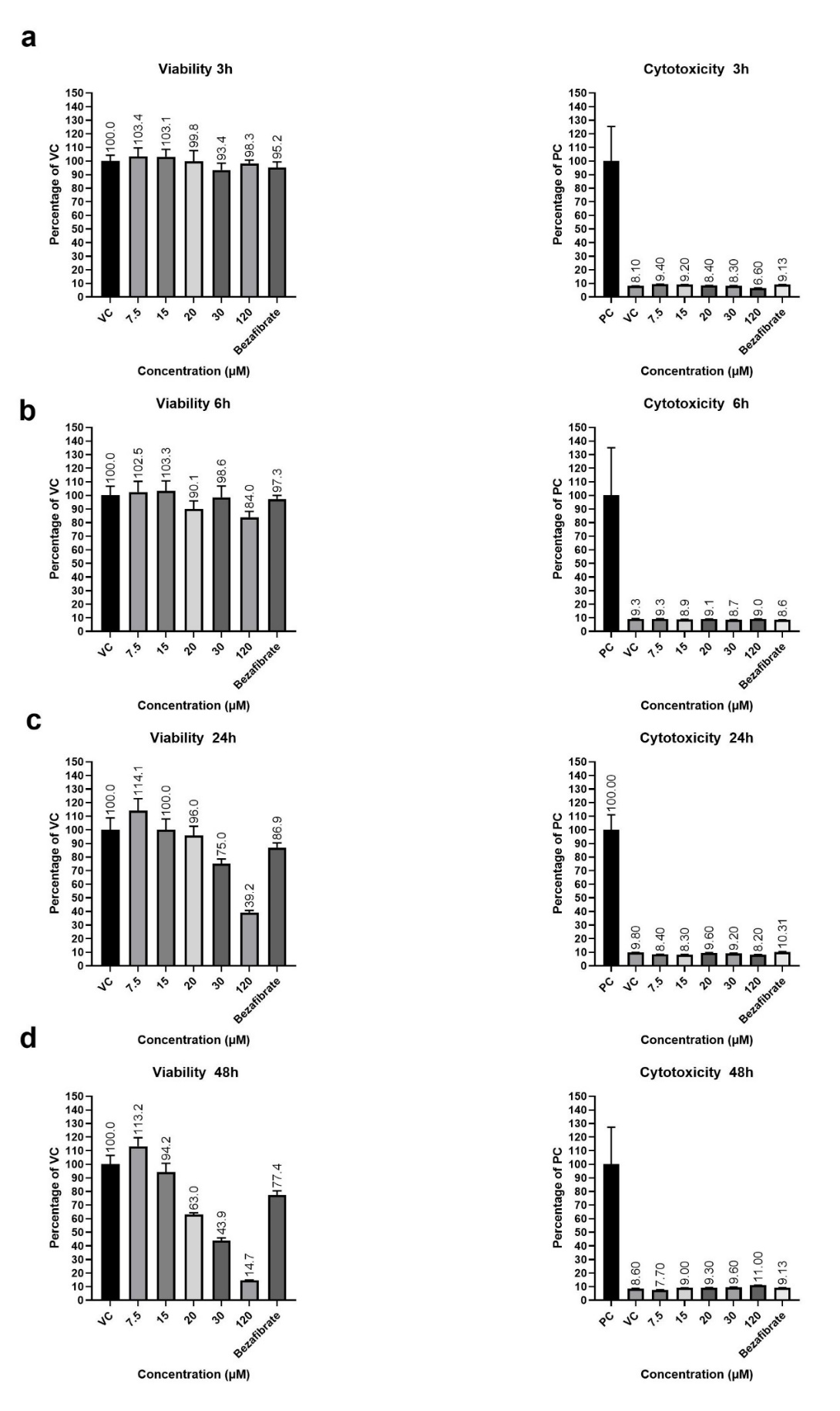


**Suppl. Fig.3 Cell viability and cytotoxicity testing in metabolomics experiments**. Cell viability ATP content-based assay (CellTiter-Glo®) and membrane impermeable DNA-binding dye-based cytotoxicity assay (CellTox™ Green) were carried out in parallel with metabolomics experiments in plates handled and treated exactly as the ones used for metabolite profiling. n=6. a) 3h, b) 6h, c)24h, d) 48h. Values are presented as percentage of vehicle controls (VC) for CellTiter-Glo® and as percentage of positive control (PC) (lysis buffer) for CellTox™ Green. Bezafibrate 1000µM was used as a positive control in each experiment.


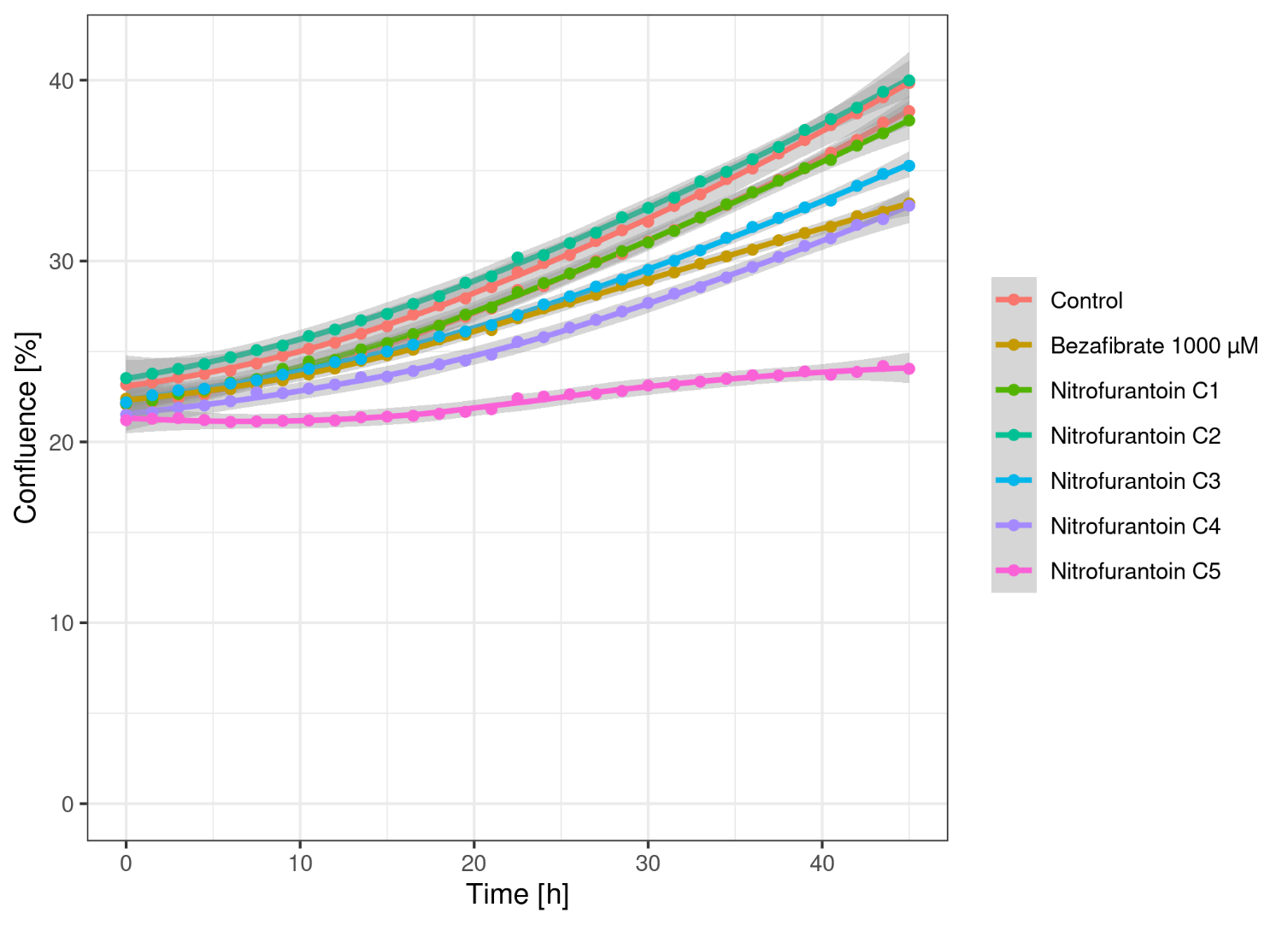


**Suppl. Fig .4 Cell confluence during 48h of nitrofurantoin exposure at different concentrations.** HepG2 were exposed for 48h to five different cocentrations of nitrofurantoin (C1:7.5µM, C2:15µM, C3:30µM, C4:60µM, C5:120µM) Cell confluence was minitored in realtime during the time of the assay. VC: vehivle control (0.5% DMSO), bezafibrate 100µM was used as a possitive control. n=6. Grey ribbon represents the confidence interval for each curve.


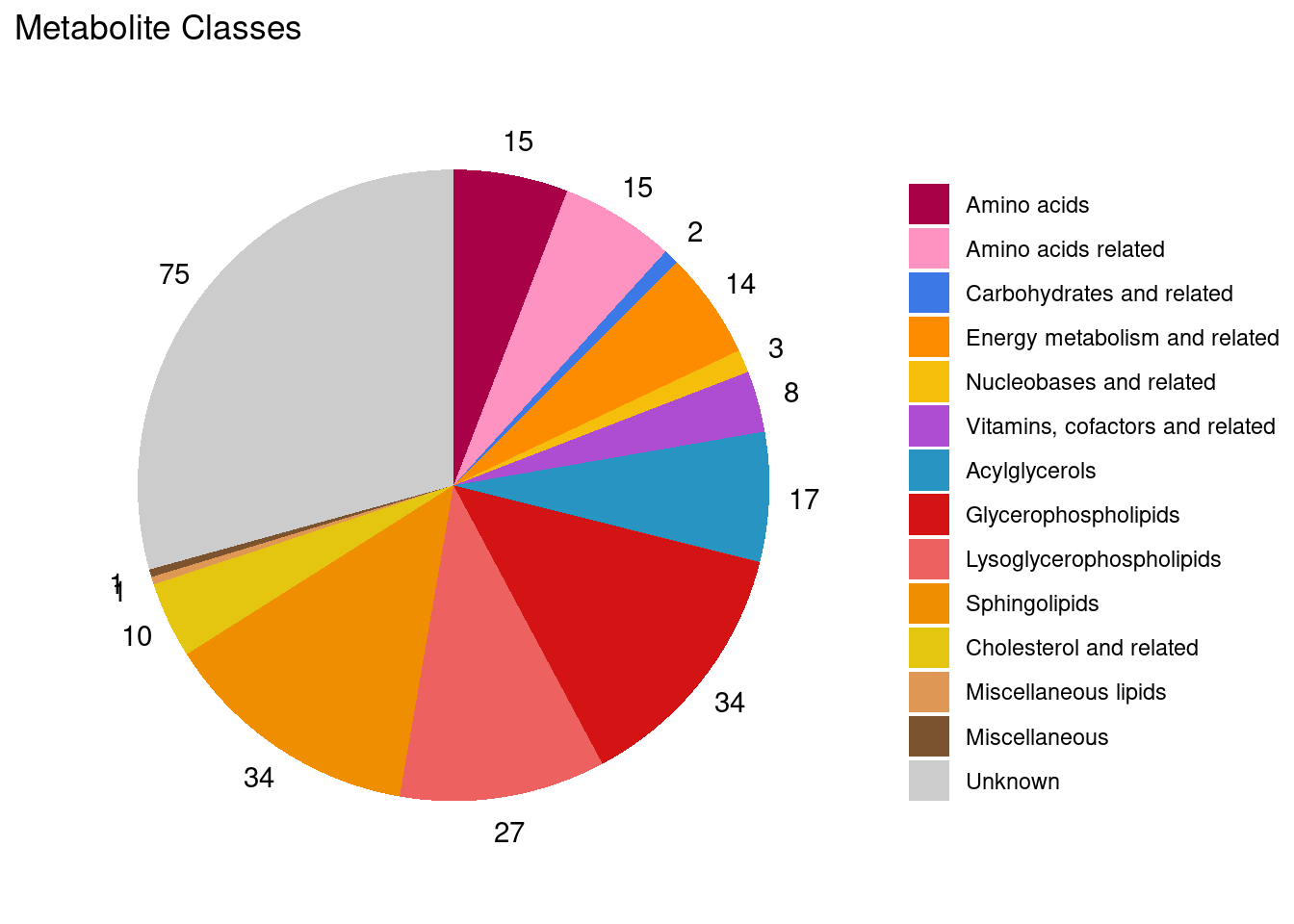


**Suppl. Fig 5 Measured metabolite classes.** Pie chart of the number of metabolites measured per ontology class. A total of 256 unique analytes were measured in this study.

| **Time point** | **Pools** | **Controls** |
| --- | --- | --- |
| 03 h | 9.2% | 12.3% |
| 06 h | 10.0% | 10.6% |
| 24 h | 9.7% | 11.3% |
| 48 h | 10.3% | 13.3% |

**Suppl. Fig.6 Experimental variability and reproducibility.** the variance of every log-transformed metabolite for both pooled samples (technical replicates) and control samples was calculated. These variances were back transformed to linear scale, yielding a relative standard deviation.

a)

b)

**Suppl Fig. 7 Enrichment analysis of significantly altered metabolites by ontology class after nitrofurantoin treatment.** The distribution of the 256 measured metabolites across the ontology classes is provided in the column “# metabolites”. The number of metabolite changes are shown for each metabolite ontology class. Numbers yellow represent that a treatment caused a significant (p-value<0.05) enrichment in an ontology class. C1 to C5: nitrofurantoin concentrations. a) changes over time, b) over concentration.

**Suppl Fig. 8a Metabolome changes induced by nitrofurantoin treatment over different time points.** Heatmap of statistically significantly (p<0.05) altered metabolites after nitrofurantoin treatment. Red represents significantly upregulated metabolites and blue represents significantly downregulated metabolites as compared to controls. Strong color, p<0.01; medium color, 0.01 ≤ p < 0.05; light color, 0.05 ≤ p < 0.10; bold typeface, > 2-fold change (increase or decrease). Sample numbers are n=6 per condition, n=12 for controls. a) Individual metabolite changes over time, b) individual metabolite changes over concentration.

**Suppl Fig. 8b Metabolome changes induced by nitrofurantoin treatment over different concentrations.** Heatmap of statistically significantly (p<0.05) altered metabolites after nitrofurantoin treatment. Red represents significantly upregulated metabolites and blue represents significantly downregulated metabolites as compared to controls. Strong color, p<0.01; medium color, 0.01 ≤ p < 0.05; light color, 0.05 ≤ p < 0.10; bold typeface, > 2-fold change (increase or decrease). Sample numbers are n=6 per condition, n=12 for controls. a) Individual metabolite changes over time, b) individual metabolite changes over concentration.


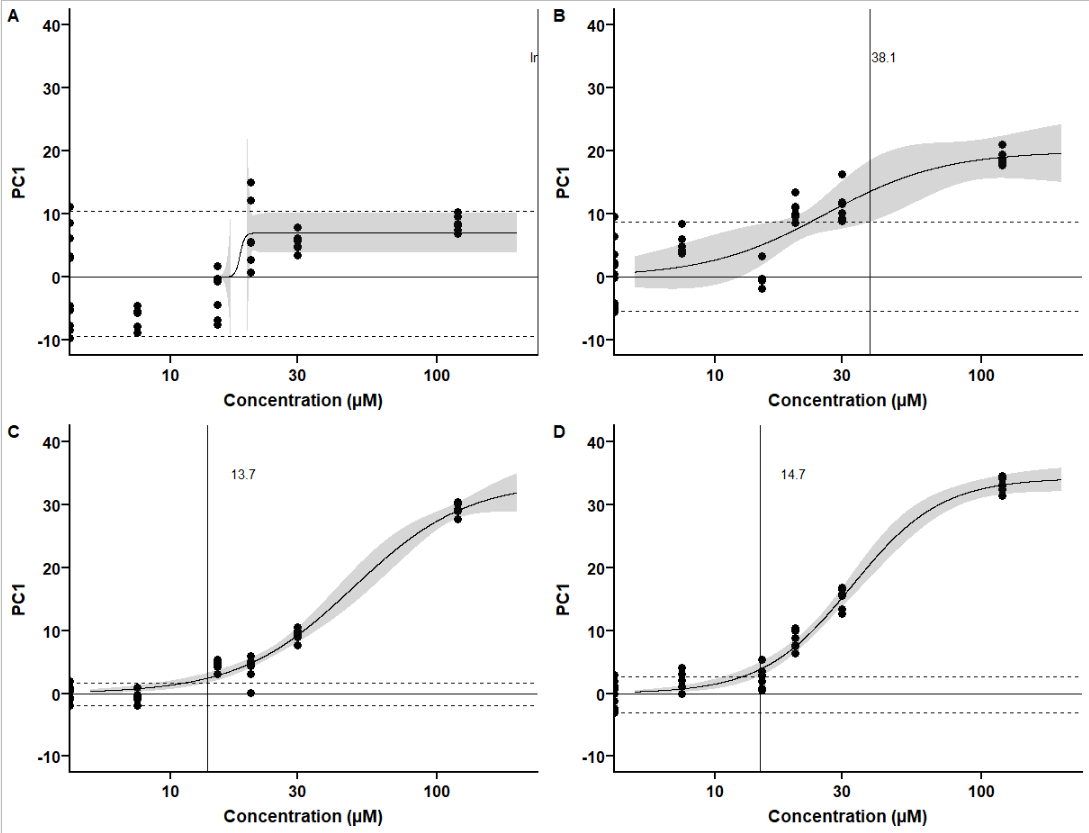


**Suppl Fig. 9 Point of departure (PoD) derivation from metabolomics data at different time points. Global metabolite changes as estimated by principal component analysis (PCA) exhibit exposure concentration dependency.** For the PoD derivation, a concentration-dependent response was fitted based on PC1 values obtained from the PCAs of a) 3 hours, b) 6 hours, c) 24 hours, and d) 48 hours nitrofurantoin treated cells at five concentrations. PC1 values for each sample were plotted against the test concentration and a 3-parameter log-logistic model was fitted through the data. A confidence interval of 95 % was used for the dose-response curve (denoted by the grey ribbon). The spread of controls is marked by the horizontal dashed lines, which represent the 2.5 % and 97.5 % quantiles; the mean is represented as horizontal solid line. The point of departure (PoD), marked by a vertical solid line, marks the concentration at which the confidence interval of the curve surpasses the corresponding quantile of the controls for the first time.
